# A probit- log- skew-normal mixture model for repeated measures data with excess zeros, with application to a cohort study of paediatric respiratory symptoms

**DOI:** 10.1186/1471-2288-10-55

**Published:** 2010-06-14

**Authors:** Sadia Mahmud, WY Wendy Lou, Neil W Johnston

**Affiliations:** 1 Department of Community Health Sciences, The Aga Khan University, Stadium Road, P O Box 3500, Karachi 74800 Pakistan; 2 Dalla Lana School of Public Health, University of Toronto, Toronto, Canada; 3 Firestone Institute for Respiratory Health, St Joseph's Healthcare and McMaster University Department of Medicine, Hamilton, Ontario, Canada

## Abstract

**Background:**

A zero-inflated continuous outcome is characterized by occurrence of "excess" zeros that more than a single distribution can explain, with the positive observations forming a skewed distribution. Mixture models are employed for regression analysis of zero-inflated data. Moreover, for repeated measures zero-inflated data the clustering structure should also be modeled for an adequate analysis.

**Methods:**

Diary of Asthma and Viral Infections Study (DAVIS) was a one year (2004) cohort study conducted at McMaster University to monitor viral infection and respiratory symptoms in children aged 5-11 years with and without asthma. Respiratory symptoms were recorded daily using either an Internet or paper-based diary. Changes in symptoms were assessed by study staff and led to collection of nasal fluid specimens for virological testing. The study objectives included investigating the response of respiratory symptoms to respiratory viral infection in children with and without asthma over a one year period. Due to sparse data daily respiratory symptom scores were aggregated into weekly average scores. More than 70% of the weekly average scores were zero, with the positive scores forming a skewed distribution. We propose a random effects probit/log-skew-normal mixture model to analyze the DAVIS data. The model parameters were estimated using a maximum marginal likelihood approach. A simulation study was conducted to assess the performance of the proposed mixture model if the underlying distribution of the positive response is different from log-skew normal.

**Results:**

Viral infection status was highly significant in both probit and log-skew normal model components respectively. The probability of being symptom free was much lower for the week a child was viral positive relative to the week she/he was viral negative. The severity of the symptoms was also greater for the week a child was viral positive. The probability of being symptom free was smaller for asthmatics relative to non-asthmatics throughout the year, whereas there was no difference in the *severity *of the symptoms between the two groups.

**Conclusions:**

A positive association was observed between viral infection status and both the probability of experiencing any respiratory symptoms, and their severity during the year. For DAVIS data the random effects probit -log skew normal model fits significantly better than the random effects probit -log normal model, endorsing our parametric choice for the model. The simulation study indicates that our proposed model seems to be robust to misspecification of the distribution of the positive skewed response.

## Background

Zero-inflated data is frequently encountered in health science studies and is characterized by the occurrence of "excess" zeros that more than a single distribution can explain. There is a considerable amount of literature dealing with the problem of zero-inflated count data such as Zero Inflated Poisson (ZIP) or Zero Inflated Binomial (ZIB) mixture models, and their extension to clustered or longitudinal data structures [1-7]. The early research on modeling zero-inflated continuous data was reported in econometrics literature [8]. Tobin [9] proposed the "Tobit" model assuming an underlying normally distributed variable whose non-positive values were considered as unobserved. In the Tobit model the observed zeros were treated as the unobserved non-positive values of the underlying variable that have been left-censored, and a linear regression model with normally distributed errors was suggested. Thereby the same stochastic process and regression parameters determined whether the response was zero or positive, as well as the magnitude of the positive response. Cragg [10] proposed a "two-part" model for semi-continuous data that separately modeled the dichotomous nature of the response (zero versus positive values), and the magnitude of the positive values respectively. Cragg suggested the probit link for modeling the binary part and a truncated normal density for the positive values, allowing a different set of covariates to be associated with the probability of having a non-zero response and the magnitude of the positive response respectively. Duan et al. [11] suggested a logit/lognormal coupling for the two part model for semi continuous data, and applied this model to demand for medical care. Moulton and Halsey [12] generalized the two-part model with logit/lognormal coupling by incorporating interval censoring, implying that the observed zeros were either a realization of the true zero point distribution or observations from the distribution of the positive outcome observed as zero due to detection limits. Heckman [13,14] extended the Tobit model to a two-part model referred to as the sample selection model that assumed an underlying bivariate normal error. Duan et al [11,15] pointed out that this model has poor numerical and statistical properties. Further discussion and references regarding sample selection model and its comparison with the two-part model are provided by Min and Agresti [8].

Olsen and Schafer [16] and Tooze et al [17] extended the two-part logit- lognormal mixture model, proposed by Duan et al. [11] for cross-sectional data, to repeated measures data by including two subject specific random effects in the logit and log-normal components respectively. These authors assumed that the random effects follow a bivariate normal distribution, and allowed for the two random effects to be correlated. Recently some additional work has been reported in literature extending mixture models for a continuous outcome with a discrete component to clustered data. Li et al [18] presented a zero-inflated log-normal model that takes hierarchical clustering structure of a data into account; they incorporated nested random intercepts in the linear predictors of the logit and lognormal model components respectively, assuming the random effects are independently and normally distributed. Liu et al [19] proposed a multi-level two-part random effects logit-lognormal model; two nested random effects were included in each part to model the nested clustering structure in a data, assuming the respective random effects in the two parts followed a bivariate normal distribution. More recently Su et al [20] showed that bias can be induced for regression coefficients when random effects are truly correlated but misspecified as independent in a 2-part mixed model.

The positive part of a zero-inflated continuous variable is often skewed to the right, logarithmic transformation had been suggested to correct for the skewness. Although Olsen and Schafer [16] allowed a more general transformation (a monotone increasing function) that would make the positive component approximately Gaussian, they only used a log transformation in the illustrative example reported in their paper and did not discuss the choice of the adequate transformation. The customary statistical approach of applying a log transformation in setting of right skewness is ad hoc, and may or may not optimally account for distributional characteristics of the data under study. As a referee noted the log transformation may often over-transform the data making the distribution skewed in the opposite direction. In an attempt to remedy this problem Chai and Bailey [21] extended the cross-sectional two-part model by suggesting a skew-normally distributed error in the regression equation for log-transformed positive values, and proposed a probit/log-skew normal mixture model for cross-sectional data. The skew-normal distribution accommodates asymmetry in a more flexible manner, and can model both positively or negatively skewed data (depending on the sign of the skewness parameter) reducing to the normal distribution when the skewness parameter is zero. Tooze et al [22] and Kipnis et al [23] suggested a different remedy to deal with the problem of skewness of the positive responses in the two-part model for longitudinal semi-continuous data. They introduced the Box-Cox transformation of the positive responses so that on the transformed scale the within subject error and the subject specific random effect in the regression model of the positive part were approximately normally distributed. The normality transformation was done within the modeling step so that the positive responses were transformed to normality conditionally on the covariates in the model, and the Box-Cox transformation parameter was estimated along with the regression parameters in the maximum likelihood procedure. When adopting the Box-Cox transformation approach one has to make an assumption that such a transformation does exist. In the present communication we adopt the approach suggested by Chai and Bailey [21] to model the positive part of semi continuous data using the skew-normal distribution. In the Discussion section we will comment on the comparison of the Box-Cox transformation approach with our present model.

In this paper we present an extension of the cross-sectional two-part Bernoulli-log-skew-normal mixture model, suggested by Chai and Bailey [21], for longitudinal zero-inflated continuous data. We modeled the clustering structure of the data by introducing correlated bivariate normal random effects in both parts, similar to what Olsen and Schafer [16] and Tooze et al [17] did for modeling longitudinal data in the two-part model with normally distributed error for log transformed positive responses. As discussed above Chai and Bailey [21] suggested the Bernoulli-log-skew-normal model for cross-sectional data thereby presenting a more flexible approach for modeling the asymmetry of the positive responses as compared to the ad hoc log transformation. Like Chai and Bailey [21] we used the probit link to model the binary component, however a logit link can also be used. The potential of the proposed model was demonstrated through analysis of a real data from a study titled "Diary of Asthma and Viral Infections Study". In addition, by fitting a random effects probit-lognormal mixture model on the dataset, we conducted a likelihood ratio- test for the skewness parameter in the log skew normal distribution, and demonstrated that the random effects probit/log-skew normal mixture model fits better on the dataset as compared to the random effects Bernoulli-log normal model proposed in references [16-19]. Moreover, in order to assess the aptness of the proposed probit/log-skew normal mixture model we conducted a probit/log-beta regression simulation for repeated measures data.

## Methods

### Probit log-skew normal mixture model for repeated measures

Let Y_ij _be an observation from the j_th _measurement on the i_th _subject with all Y_ij _≥ 0. The probability density function of Y_ij _is:(1)

We used the probit link function for p_ij_:

where Φ is the standard normal cumulative distribution function, *X*_(1)*ij *_is the vector of explanatory variables associated with the probability of the i_th _subject being symptom free at the j_th _occasion, and *β*_(1) _is the vector of corresponding regression parameters. The positive outcome, Y_ij _> 0, was assumed to follow a skew normal (SN) distribution.

*X*_(2)*ij *_is the vector of explanatory variables associated with the severity of symptoms for the i_th _subject at the j_th _occasion, *β*_(2) _and is the vector of corresponding regression parameters. *μ,σ,δ *are the parameters of the skew normal distribution (we are setting *μ *= *X^T^*_(2)*ij *_*β*_(2) _+ *τ*_1*i*_), *δ *is referred to as the skewness parameter (see Additional File 1). The reason for modeling the positive outcome on the logarithmic instead of the original scale is to ensure positive estimation as log of negative numbers does not exist. In order to model the correlation among repeated measurements on the same subject, we included two random effects (*τ*_0*i *_, *τ*_1*i*_) in the linear predictors of the two regression model components respectively. We assumed that these random effects follow a bivariate normal distribution (BVN), that is,

*S*_11 _and *S*_22 _being the variance of the random effects in the probit and log-skew-normal components respectively and *S*_12 _being the covariance between the two random effects.

### Maximum Marginal Likelihood Estimation

Defining an indicator function, *I*_*ij *_(*I*_*ij *_= 1 if Y_ij _= 0, *I*_*ij *_= 0 if Y_ij _> 0) likelihood contribution from the i_th _subject can be expressed as follows:

where *m_i _*is the number of repeated measurements on the i_th _subject and f(*τ*_0*i*_, *τ*_1*i*_) is the joint distribution of the two random effects. We assumed that f(*τ*_0*i*_, *τ*_1*i*_) is bivariate normal.

Assuming the measurements on different subjects are independent, the likelihood to be maximized is:

where *n *is the total number of subjects in the sample. In the Maximum Marginal Likelihood Estimation approach integration over random effects is approximated by numerical integration. We used Gaussian quadrature to obtain the marginal likelihood and employed Double Dogleg optimization method to maximize the likelihood. This optimization technique combines the concept of the Trust Region and Quasi- Newton methods and works well for medium to moderately large optimization problems [24-26].

### Diary of Asthma and Viral Infection Study

Respiratory viral infections (RVI), most commonly of rhinovirus, have been found to coincide with the majority of children's asthma exacerbations throughout the year, including the post summer vacation epidemic periods, in both community and hospital based studies [27-30]. Children admitted to hospital for wheezing have been shown to have a significantly higher rate of RVI [28,29]. Furthermore, asthma exacerbations in children are highly cyclic and follow predictable seasonal patterns [30]. The 'Diary of Asthma and Viral Infection Study (DAVIS)', a 12 month, cohort study was conducted at McMaster university to monitor infection and respiratory symptoms including asthma exacerbations in children aged 5-11 years with and without asthma. The study objectives included investigating the response of respiratory symptoms to RVI in children with and without asthma over a one year period. The study period was the 2004 calendar year. Respiratory symptoms were recorded daily using either an Internet or paper-based diary. Changes in symptoms were assessed by study staff and led to collection of further information, the use of spirometry and collection of nasal fluid specimens for virological testing. Virological testing was conducted using polymerase chain reaction techniques as previously described [31].

The study was designed and executed by academic investigators (with Neil W Johnston as the principal investigator, PI) and was approved by the Research Ethics Board of St. Joseph's Healthcare, Hamilton (R.P. #03-2195). Written informed consent for children to participate was obtained from parents of all subjects and assent from appropriately aged children. The raw data is accessible only to the PI and the research team, as was approved by the Research Ethics Board. Individuals who wish to have access to the data for replicating the study results are advised to contact the PI for necessary Research Ethics Board (or Institutional Review Board) approval.

Six lower respiratory tract (LRT) symptoms (cough during the day, cough during the night, wheeze, difficulty breathing or shortness of breath during the night, difficulty breathing or shortness of breath during the day and breathing problems interfering with child's regular activities during day) were categorised by subjects on a 5 point scale from 0 (none) to 4 (very severe). Overall daily LRT symptom scores were determined by summing the six LRT symptom scores, emulating the approach taken in a previous study [27]. For most of the subjects many daily LRT scores were zero leading to sparse data, hence daily LRT scores were aggregated to give weekly average LRT scores. This was done by writing a SAS macro that executed PROC EXPAND for each subject. PROC EXPAND can change the frequency of a single time series such as conversion from daily measurements to weekly or monthly averages or totals. The weekly interval was defined as Sunday to Saturday, giving a total of 51 weeks for the year 2004. If a daily measurement was missing for a given week for a subject, the missing value was replaced by the weekly average. If more than two measurements were missing for a week, the weekly average was treated as a missing value.

Data for 190 subjects (135 asthmatics and 55 non-asthmatics) were available for the analysis. The majority of the subjects (172) had measurements for all 51 weeks, one subject had measurements for only 41 weeks, whereas 17 subjects had measurements ranging from 44 to 50 weeks. Eleven subjects entered the study later than the 1^st ^week but no later than the 8^th ^week of the year. The subjects started dropping from the study after the 41^st ^week. One hundred and eighty- six subjects had weekly LRT measurements after the 46^th ^week.

**Figure 1:**
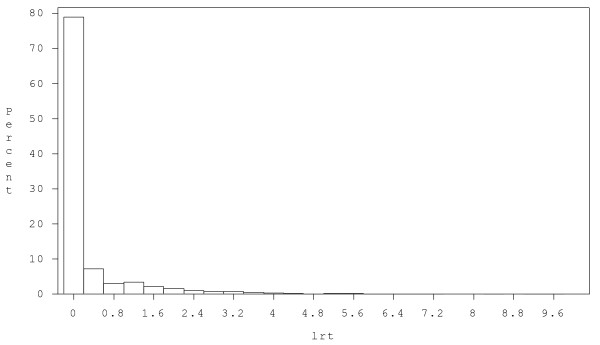
**Histogram of weekly average LRT scores**.

A histogram of weekly average LRT scores indicated that about 75% scores were zero and the positive scores seemed to be represented by a continuous skewed distribution (Figure 1). The zeros correspond to the absence of respiratory symptoms during a week, and the positive scores measured the severity of the respiratory symptoms when present. In order to account for the excess zeros and repeated measurements on each subject over the weeks of the year, we propose the mixture model (1) that first considers the response as a dichotomous variable (zero versus greater than zero), and then models the positive response using log skew normal distribution. The clustering structure of the data is modeled by the two correlated random effects in each part. The regression analysis was aimed at investigating the relationship of children's respiratory symptom scores with viral infection status throughout the year 2004, adjusting for their asthmatic status, age and sex.

The mixture model (1) was fitted using PROC NLMIXED on SAS (see SAS codes in Additional File 2). We started with fitting a main effect model with asthmatic/non-asthmatic status, child's sex and age, week of follow-up and viral infection status as independent variables in both model components (-2LL = 14181, BIC = 14270, AIC = 14215). The variable "week" was coded as week = 1 corresponding to the 1^st ^week of January (starting 4^th ^January that was the 1^st ^Sunday of January 2004), week = 2 to the 2^nd ^week and so on. Viral infection status, being a time-dependent variable, was defined as positive for a week if the subject had any respiratory virus detected during that week, negative otherwise; it was coded as "1" if the subject was viral positive and "0" if viral negative for a given week. Initially age and week of follow-up were modeled as linear continuous variables. Initial values of parameters were taken from a probit-log skew normal mixture model without random effects (that converged more quickly). In addition the initial parameter values for the variance and covariance of the random effects were set as s_11 _= 0.5, s_22 _= 0.5, s_12 _= 0. Generalized Additive Model (GAM) approach was used to examine the scale of continuous predictor variables, age and week of follow-up, with reference to their regression relationship with weekly LRT scores. GAM is a non-parametric smoothing technique for exploring the scale of an independent continuous variable for a regression model [32]. The exploratory GAM analysis indicated a quadratic scale for both age and week (more pronounced for week). Fitting the mixture model by including square terms for age and week in both model components gave -2LL = 14069, BIC = 14179, AIC = 14111. Wald p-values that were employed for preliminary screening indicated that the square terms for age could be removed from the model. Fitting the mixture model again with week of follow-up modeled as quadratic and age as linear in both model components led to -2LL= 14069, BIC= 14169, AIC= 14107. Hence based on the log-likelihood, AIC and BIC criteria the week of follow-up was modeled as quadratic, whereas age was modeled as linear in both model components respectively. Next all possible two-way interactions were included in both mixture model components (-2LL = 14037, BIC = 14241, AIC = 14115). Using the Wald p-values of the interaction terms for preliminary screening we obtained the final model (- 2LL = 14056, BIC = 14166, AIC = 14098) including two interactions that were both statistically and biologically significant (Table 1).

**Table 1:** Probit/log-skew normal model for weekly LRT scores. Parameter estimates (standard errors) n= 190.

	Final Model (LRT)	
	**probit**	**log-skew-normal**
		
β_0_	-0.635(0.285)**	0.298(0.263)
β_asthamatic_	0.598(0.104)***	0.096(0.089)
β_male_	0.024(0.078)	-0.129(0.076)*
β_age_	-0.027(0.031)	6.2e-3(0.028)
β_week_	-0.050(0.005)***	-3.0e-3(0.008)
β_week*week_	8.5e-4(0.9e-4) ***	3.5e-4(1.0e-4)***
β_virus_	2.459(0.126)***	0.744(0.057)***
β_age*week_	-	-2.0e-3(0.7e-3)***
β_asthamatic*week_	-6.4e-3(2.6e-3)**	-
σ		0.666(0.049)***
δ		-0.952(0.100)***
s11	1.023(0.183)***	
s22		0.214(0.041)***
s21		0.267(0.077)***
-2 Log Likelihood	14056	
AIC	14098	
BIC	14166	
* p-value < 0.10	** p-value < 0.05	*** p-value < 0.01

### Simulation Study

We carried out a simulation study to assess the performance of the proposed random effects probit log-skew normal model, if the underlying distribution of the positive response is different from log-skew normal. For the simulation study we generated repeated measures data from a probit log- beta model stated as follows:(2)

where *x_ij _*is a binary time dependent predictor variable (similar to the viral infection status in the DAVIS data, *x_ij _*= 1 if i_th _subject is viral positive for j_th _week, *x_ij _*= 0 if negative). *ε_ij _*follows a beta distribution with probability density:

where *α *> 0, *β *> 0 are the parameters of the beta distribution and  is the beta function. A random variable *Y *follows a four parameter beta distribution if:

where *ε *˜ *BETA*(*α, β*) and parameters Θ and (Θ + *scale*) define the minimum and maximum values of *Y *respectively. Hence in model (2) log (Y_ij _| Y_ij _> 0) follow the four parameter beta distribution with Θ = (*α*_1 _+ *β*_1_*x_ij _*+ *τ*_1*i*_). *τ*_0*i *_and *τ*_1*i *_are the subject specific random effects such that

The rationale for selecting the beta distribution to model the positive response in our simulation study was that, similar to the skew normal distribution, it can be positively (*β *>*α*) or negatively (*β *<*α*) skewed, as well as symmetric (*β *= *α*). The skewness of the beta distribution is:

We generated 50 repeated measurements corresponding to each week for each of 200 independent clusters (subjects) and assigned the following values for the model parameters, *α*_0 _= -1, *β*_0 _= 2.5, *α*_1 _= - 20, *β*_1 _= 0.75, *s*_11 _= 1, *s*_22 _= 0.2, *s*_21 _= 0.2, *scale *= 30. For each

subject at every week the time dependent binary covariate *x_ij _*was generated as a Bernoulli variable with probability = 0.03 (of all weekly average LRT scores in DAVIS data for about 3% viral infection variable was positive). The true values of *α*_1 _parameters and *scale *were specified so that the minimum and the maximum values of (Y_ij _| Y_ij _> 0) are approximately equal to e^-20 ^≈ 0 and e^(-20 +30) = ^e^10 ^(that is a very large number) respectively, thereby simulating a situation where the positive outcome can be considered as a continuous variable bounded below at zero.

We considered two scenarios with respect to specifying the parameters of the beta distribution, (i) a negatively skewed beta distribution (*β *= 70, *α *= 130, *skewness *= -0.0883) and (ii) a symmetric beta distribution (*β *= *α *= 100, *skewness *= 0). For each of the two scenarios 200 datasets were generated from model (2) and probit log-skew normal model (1) was fitted on each dataset. We also did some simulation runs generating data from a positively skewed beta distribution (*β *= 130, *α *= 70, *skewness *= -0.0883) and fitted random effects probit-log-skew normal model.

## Results

### Analysis of DAVIS

**Figure 2:**
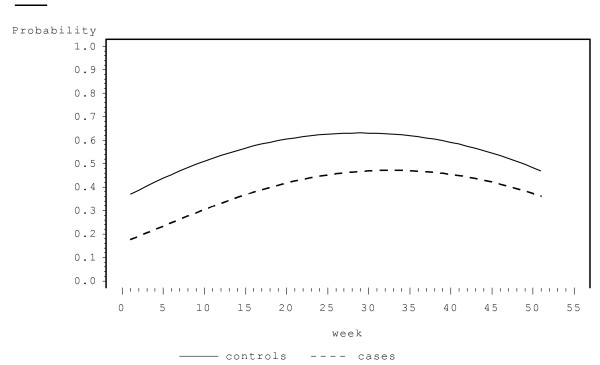
**Probability of being LRT symptom free versus the week of the year**.

The final fit of the mixture model (1) to DAVIS data is reported in Table 1. The covariates significantly associated with the probability of having no LRT symptoms were asthmatic/non-asthmatic status, week of follow-up and viral infection status (probit component). The highly significant positive estimate of β_virus _indicates that for the week a child was viral positive, the probability of being LRT symptom free was much less than that for the week the child was viral negative (p-value < 0.0001, beta = 2.459). The variable week was modeled as quadratic in the linear predictor of the regression model of the probit component. Moreover, there was a significant interaction between the asthmatic/non-asthmatic status and (the linear term of) week of follow-up (Wald p-value = 0.0150). We also examined the interaction of asthmatic/non-asthmatic status with the quadratic term in week (that is asthmatic*week*week) but that was insignificant based on the Wald, likelihood, AIC and BIC criteria. The probability of being LRT symptom free was lower in the beginning of the year, increased from January to August, and after that decreased until December (Figure 2). The association between the probability of being symptom free and the asthmatic/non-asthmatic status can be clearly seen from Figure 2; the probability of being symptom free is smaller for asthmatics relative to non-asthmatics throughout the year, the difference being more pronounced in the beginning of the year.

**Figure 3:**
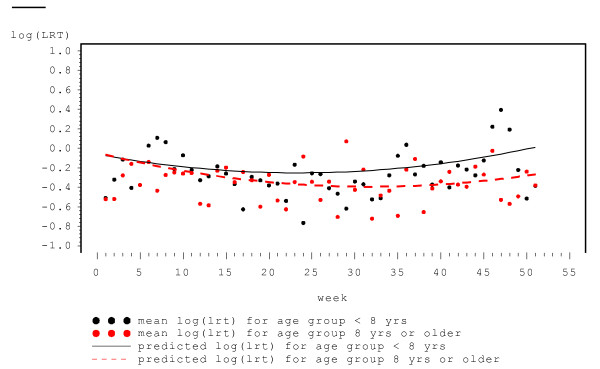
Log (LRT > 0) versus the week of the year

**Figure 4:**
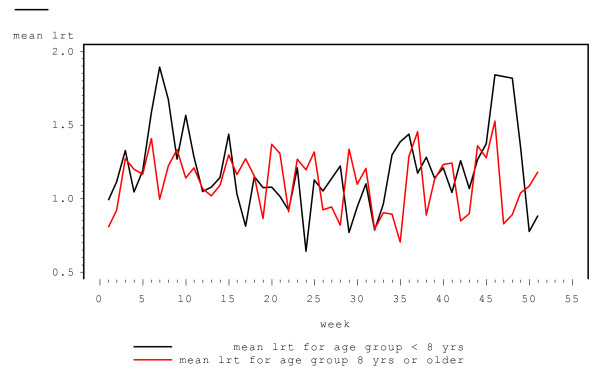
Mean LRT > 0 versus the week of the year

Covariates associated with the *severity *of LRT symptoms were subject's age and sex, viral infection status and week of follow-up (log skew normal component). There was no significant difference in the severity of the LRT symptoms between asthmatic and non-asthmatic children (p-value = 0.2758). For the week a child was viral positive the severity of LRT symptoms was significantly greater than for the week he/she was viral negative (p-value < 0.0001, beta = 0.744). There was some marginal evidence that the severity of LRT symptoms was lesser for the male relative to the female children (p-value = 0.0938, beta = -0.1286). As for the probit component, the quadratic term for week was significant in the log-skew normal component. Moreover, there was a significant interaction between the age of a child and (the linear term of) the week of follow (p-value = 0.0069). We also examined the interaction of age with the quadratic term in week (that is age*week*week), however that was insignificant based on the Wald, likelihood, AIC and BIC criteria. In Figure 3 we plot predicted values of log(LRT > 0) from the fitted model versus week of the year for two age groups, < 8 years (mean age) and ≥8 years, along with mean log(LRT > 0) values at each week computed from the data. (In Figure 4 mean LRT > 0 scores on the original scale, computed from the data, are plotted versus week of follow-up). The severity of LRT symptoms was higher in the beginning of the year, decreased in summer and increased again by the end of the year. The severity of LRT symptoms was higher for children younger than 8 years relative to older children, and seemed to exhibit a more pronounced seasonal pattern.

From the model fit in Table 1 we note that the Wald p-value for the skewness parameter (δ) was highly significant (p-value < 0.0001, estimate of δ = -0.952). This suggests the importance of using the log-skew-normal distribution to model the positive response for this data, using the log-normal distribution would be inadequate. We also conducted the likelihood ratio test for testing the hypothesis H_0_: δ = 0 by fitting a probit log-normal model; chi-square test statistic, *x^2 ^*(*df *= 1) = 10, p-value < 0.005 indicating significance of the skewness parameter (δ). In addition we note that for the probit-log-normal mixture model AIC = 14106 and BIC = 14171, these criteria also suggest that the probit-log- skew normal mixture model reported in Table 1 is superior to probit log-normal model. We note the sign of the estimate of the skewness parameter indicating a negatively skewed distribution for the log of positive LRT scores. We would like to point out here that for certain parameterization of skew normal distribution the Fisher information matrix is singular when asymmetry parameter is equal to zero. This leads to difficulties regarding asymptotic distributions for maximum likelihood estimators and likelihood ratio statistic [33]. However, for alternative parameterization of skew normal distribution these difficulties are resolved, and the likelihood function exhibits a more regular behaviour without a stationary point when the asymmetry parameter is equal to zero [33]. In our model we used the skew normal parameterization suggested by Sahu et al [34] that was also employed by Chai and Bailey [21] in their probit -log- skew normal mixture model for zero-inflated continuous cross-sectional data. Chai and Bailey [21] conducted and reported the likelihood ratio test for testing the null hypothesis: asymmetry parameter (for the skew-normal distribution) = 0.

Moreover, the Wald p-values for the variance of the random effects (s_11_, s_22_) in the two model components respectively were highly significant (p-value < 0.0001) indicating the random effects were needed in the model to account for the correlation among measurements on the same subject. The significant *positive *covariance (s_21_) between the two random effects has an intuitively appealing interpretation; the higher the probability of a subject of being positive for LRT symptoms, the greater the severity of LRT symptoms.

### Results of the simulation study

The results of the simulation study are presented in Table 2. In the simulation study we generated data from random effects probit log-beta model (2) and fitted random effects probit log-skew normal model (1). In Table 2 we report the bias and mean square error for the estimated values of the intercept (in the probit component), regression coefficients corresponding to the time dependent binary variable, *x_ij _*and the variance and covariance of the random effects in the two model components respectively from the simulation runs. We note that the regression coefficient corresponding to *x_ij _*in the continuous part, for both log-skew-normal and log-beta model, is the difference in the expected value of log(Y_ij _| Y_ij _> 0) when *x_ij _*= 1 versus *x_ij _*= 0 and hence is comparable. However, the intercepts in the log-skew-normal and log-beta models are not comparable. As discussed in the Methods section, we considered two scenarios with respect to specifying the parameters of the beta distribution (i) a negatively skewed beta distribution, and a (ii) symmetric beta distribution. For both these scenarios the simulation results indicate that the estimates seem to be unbiased, particularly for the regression coefficients corresponding to the time dependent predictor variable in both model components. This suggests that the probit-log skew normal model performs reasonably well, as the primary goal of the simulation study was to assess the ability of the model to estimate the affect of an explanatory variable on the response.

**Table 2:** Simulation Results from 200 simulation runs. Fitted model is probit-log-skew-normal.

PROBIT component	β < α ^1 ^ (negatively skewed)		β = α ^2 ^ (symmetric)	
	
	Bias	MSE ^4^	Bias	MSE
Intercept (-1)^3^	0.0303	0.0128	0.0054	0.0116
β_Viral Infection _(2.5)	-0.0186	0.0130	-0.0064	0.0112
s11 (1)	-0.0927	0.0243	-0.0914	0.0235

**Log-beta component**				
Intercept (-20)				
β_Viral Infection _(0.75)	0.0004	0.0050	-0.0090	0.0055
s22 (0.2)	-0.0059	0.0010	-0.0085	0.0012
s12 (0.2)	-0.0167	0.0028	-0.0199	0.0033

1. β = 70, α = 130

2. β = α = 100

3. Numbers in parentheses refer to true parameter values

4. Mean Square Error (MSE)

For scenario (i) where we generated the datasets from a negatively skewed beta distribution, the mean (standard deviation) of estimates of δ (the skewness parameter in the probit-log-skew normal model) was -0.7031 (0.1951) and for scenario (ii) corresponding to a symmetric beta distribution, the mean (standard deviation) of estimates of δ was -0.3171 (0.2760) from the 200 simulation runs. This suggests that the log-skew normal model correctly identified the *negatively *skewed as well as the symmetric distribution; for the former the mean estimated value of δ was negative, and for the latter though the mean estimated value was somewhat negative but does not appear to be significantly different from zero due to the large standard deviation. We also did some simulation runs generating data from a positively skewed beta distribution (*β *= 130, *α *= 70, *skewness *= 0.0883) and fitted probit-log-skew normal model that gave similar results.

## Discussion

In this communication we have presented a probit/log-skew-normal mixture model for continuous repeated measures data with a discrete component at zero. We modeled the clustering

structure of the data by including two random effects in the probit and log-skew-normal model components respectively, assuming the random effects follow a bivariate normal distribution. In case of longitudinal data structure, in addition to random intercepts it may be of interest to include a random slope for time in the continuous component as suggested by Su et al [20]. This can be done through a straightforward extension of the model we proposed by including a random slope for time in the log-skew normal component, assuming the three random effects follow a trivariate normal distribution. Recent research has focused on the impact of misspecification of random effects distribution on the maximum likelihood estimates for generalized linear mixed models (GLMM). For the case of linear mixed models (that correspond to the identity link function for GLMM) the parameter estimates are rather robust with respect to deviation from normality of random effects. However, for random-intercept logistic models the estimates of the mean structure parameters can have substantial bias upon misspecification of random effects distribution in case of large random effects variance [35]. In our present analysis the maximum likelihood estimates of the variances of random intercepts in the two parts are rather small (of the order of magnitude of 1 in the binary and 0.2 in the continuous component) thereby suggesting that the potential bias in the estimates of fixed effects in case of misspecification of random effects distribution could be small. However, there can be considerable bias in the estimate of variance components in case of misspecification of the random effects distribution, thereby making it difficult to distinguish between the small or large variance scenarios [35]. This suggests the need for further research to investigate the impact of misspecification of random effects distribution on the estimates of fixed and random effects in a two-part mixture model for semi-continuous data. This research will be particularly relevant as the continuous model component constitutes a non-linear mixed model. Investigating the impact of the distribution of random effects on parameter estimation in mixture models for clustered semi continuous data will be taken up as future research.

For longitudinal data, subject specific random effect models account for the correlation among measurements on the same subject through the concept of heterogeneity among subjects; some subjects are intrinsically high responders, others low-responders. However, serial correlation models time varying stochastic process within a subject [36]. In our proposed random effects mixture model, serial correlation can be incorporated by including a lagged response variable as a predictor variable in the model [37].

The potential of the proposed model was demonstrated through analysis of a real dataset from DAVIS. The response variable of interest was the weekly average LRT symptom score; about 75% of these scores were zero and the positive scores formed a skewed distribution. We assumed that the zeros correspond to 'true zeros' indicating absence of any LRT symptoms. This assumption seems reasonable in the context of LRT symptoms reported by subjects in DAVIS, where zero scores correspond to "No symptoms". Incorporating interval censoring in a zero-inflated mixture model implies that the observed zeros are either a realization of a 'true zero' point distribution, or an observation from the distribution of the positive outcome observed as zero due to detection limitation [12]. The latter does not seem to be relevant for the self-reported LRT symptoms in DAVIS, detection limits are usually relevant for measurements involving laboratory markers [12,21].

As discussed above, in addition to random intercepts we also included a random time slope in the log-skew normal component for the main effect mixture model for DAVIS; the estimate of the variance of the random slope, though significant, was quite small (0.0002) implying that the random effect of time did not vary substantially from subject to subject.

For the positive outcome the log-skew-normal distribution fits significantly better, for the dataset we used as an example, as compared to log-normal distribution suggested by authors in references [17-20]. For the binary component of the mixture model either a probit or a logit link can be used, authors in references [17-20] presented a logit-lognormal coupling for their mixture models. We also fitted a logit-log-skew-normal random effects mixture model on DAVIS data, however with the probit link the likelihood of the fitted model was higher than that with the logit link, though a likelihood ratio test could not be conducted as the models were not nested.

We conducted a simulation study, to assess the performance of log skew normal distribution in modeling the positive component of the response, by generating repeated measures data from a probit log-beta model, and fitting the proposed probit-log-skew normal mixture model. The results of the simulation study indicate that the probit-log-skew normal mixture model performs reasonably well in estimating the true regression parameters, when the underlying distribution of the log transformed positive response was a beta distribution. Like the skew-normal distribution, the beta distribution can be positively or negatively skewed or symmetric. The skew-normal distribution did demonstrate an ability to recognize a negatively skewed and a symmetric beta distribution correctly, though the skewness parameters for the skew-normal and the beta distribution were not directly comparable.

Finally the Box-Cox transformation approach to normally transform both the skewed positive observations of a semi continuous longitudinal outcome, and the subject specific random effects in the regression model of the positive part [22,23] seems to be a potentially interesting alternative to the random effects regression model with log-skew-normally distributed errors we have suggested in this paper. A formal comparison between these two different approaches will be taken up as future research.

## Conclusions

Mixture models offer an informative and elegant regression approach, allowing assessment of association of a potential risk factor with *both *the probability of being symptom free and the severity of symptoms for a response with clustering at zero. We proposed a probit-log skew normal mixture model for zero-inflated repeated measures data, and demonstrated its potential by analyzing real data from DAVIS. We showed that for this data probit-log skew normal mixture model fits significantly better than the Bernoulli -log normal model proposed in previous references. The probability of a child being free of lower respiratory track symptoms was lower for a week he/she was positive for viral infection relative to a week viral infection was negative. Moreover, the severity of the respiratory symptoms was greater for the week the child was viral positive. The results of our simulation study indicate that our proposed model performs reasonably well even if the underlying distribution of the positive outcome is misspecified.

## List of abbreviations

AIC: (Akaike's Information Criterion); BIC: (Bayesian Information Criterion); BVN: (Bivariate Normal Distribution); DAVIS: (Diary of Asthma and Viral Infections Study); GAM: (Generalized Additive Model); GLMM: (Generalized Linear Mixed Models); LRT: (lower respiratory tract); LL: (log-likelihood); PI: (principal investigator); RVI: (respiratory viral infections); ZIB: (Zero Inflated Binomial); ZIP: (Zero Inflated Poisson).

## Competing interests

The authors declare that they have no competing interests.

## Authors' contributions

SM conducted the analysis and simulation reported in the paper, and wrote the statistical component of the manuscript. WL supervised post doctoral research (reported in the paper) conducted by SM. NWJ provided original data and conducted the study (DAVIS) from which it was derived. He provided input for writing the introduction and methods for conducting DAVIS.

All authors read and approved the final manuscript.

## Pre-publication history

The pre-publication history for this paper can be accessed here:

http://www.biomedcentral.com/1471-2288/10/55/prepub

## Supplementary Material

Additional file 1 **Skew-Normal Distribution**. Probability Density Function of Skew-Normal Distribution. Click here for file

Additional file 2 **SAS codes**. SAS codes for implementing Maximum Marginal Likelihood Estimation of the Random Effects Probit- Log Skew Normal model and Random Effects Logit- Log Skew Normal model respectively. Click here for file
