# Supplementary material for: A probit- log- skew-normal mixture model for repeated measures data with excess zeros, with application to a cohort study of paediatric respiratory symptoms
Source: BMC Med Res Methodol. 2010 Jun 14;10:55. doi: 10.1186/1471-2288-10-55 (PMC2902491; doi:10.1186/1471-2288-10-55)
Supplement: Additional file 1 — Skew-Normal Distribution. Probability Density Function of Skew-Normal Distribution. [file 1471-2288-10-55-S1.PDF]

Skew Normal Distribution

A random variable Z follows a Skew-Normal distribution if its probability density function (pdf) is:

$$f(z) = \frac{2}{\sqrt{\sigma^2+\delta^2}}\phi(\frac{z-\mu}{\sigma^2+\delta^2})\Phi(\frac{\delta}{\sigma}\frac{z-\mu}{\sigma^2+\delta^2}), \qquad z \in R$$

where ϕ and Φ are the standard normal pdf and cdf(cumulative distribution function) respectively. δ is referred to as the skewness parameter, when δ is zero $Z \sim N(\mu, \sigma^2)$.

Expected value of Z and its variance are as follows:

$$E(Z) = \mu + \delta\sqrt{2/\pi} \qquad \text{and} \qquad Var(Z) = \sigma^2 + \delta^2(1-\frac{2}{\pi})$$
