# Supplementary material for: A probit- log- skew-normal mixture model for repeated measures data with excess zeros, with application to a cohort study of paediatric respiratory symptoms
Source: BMC Med Res Methodol. 2010 Jun 14;10:55. doi: 10.1186/1471-2288-10-55 (PMC2902491; doi:10.1186/1471-2288-10-55)
Supplement: Additional file 2 — SAS codes. SAS codes for implementing Maximum Marginal Likelihood Estimation of the Random Effects Probit- Log Skew Normal model and Random Effects Logit- Log Skew Normal model respectively. [file 1471-2288-10-55-S2.PDF]

SAS Code for implementing Maximum Marginal Likelihood Estimation of the Random Effects Probit- Log Skew Normal model

```
data data1; set data1 ;
if (lrt = .) then delete;
if lrt > 0 then loglrt = log(lrt);
else loglrt = -100;
run;
```

```
proc nlmixed data = data1  noad tech=dbldog;
parms  /* initial values of fixed effects parameters were taken from Probit- Log Skew
Normal model without random effects */ s11=0.5 s22= 0.5 s21=0;

bounds s11> 0; bounds s22> 0;
 ll=0;

eta0 = b0 + b1*asthmatic + b2*male + b3*age + b4*week1 + b5*virus +
b44*week1*week1 + b14* asthmatic *week1 + u1;

 eta1 = d0 + d1* asthmatic + d2*male + d3*age + d4*week1 + d5*virus +
d44*week1*week1 + d34*age*week1  + u2;


A1 = sqrt(sigma*sigma + delta*delta);
A = 2/A1;

p0 = CDF('normal', -eta0);

if (loglrt = -100) then p=p0;

else if (loglrt^= -100) then
p = (1-p0)*A*PDF('normal',(loglrt - eta1)/A1)*
CDF('normal', delta/sigma *(loglrt - eta1)/A1);

ll= log(p);


model loglrt ~ general (ll);
random u1 u2 ~ normal ([0,0],[s11,s21,s22]) subject = id;
run;
```

SAS Code for implementing Maximum Marginal Likelihood Estimation of the Random Effects Logit- Log Skew Normal model

```
data data1; set data1 ;
if (lrt = .) then delete;
if lrt > 0 then loglrt = log(lrt);
else loglrt = -100;
run;

proc nlmixed data = data1 noad tech=dbldog;
parms  /* initial values of fixed effects parameters were taken from Logit- Log Skew
Normal model without random effects */   s11=0.5 s22=0.5 s21=0;

bounds s11> 0; bounds s22> 0;
ll=0;

eta0 = b0 + b1* asthmatic + b2*male + b3*age + b4*week1 + b5*virus +
b44*week1*week1 + b14* asthmatic *week1 + u1;


eta1 = d0 + d1* asthmatic + d2*male + d3*age + d4*week1 + d5*virus +
d44*week1*week1 + d34*age*week1 + u2;

A1 = sqrt(sigma*sigma + delta*delta);
A = 2/A1;

p0 = CDF('logistic', -eta0);

if (loglrt = -100) then p=p0;

else if (loglrt^= -100) then
p = (1-p0)*A*PDF('normal',(loglrt - eta1)/A1)*
    CDF('normal',delta/sigma *(loglrt - eta1)/A1);

ll= log(p);


model loglrt ~ general (ll);
random u1 u2 ~ normal ([0,0],[s11,s21,s22]) subject = id;
run;
```
